# Predictive value of metabolic 18FDG-PET response on outcomes in patients with locally advanced pancreatic carcinoma treated with definitive concurrent chemoradiotherapy

**DOI:** 10.1186/1471-230X-11-123

**Published:** 2011-11-10

**Authors:** Erkan Topkan, Cem Parlak, Ayşe Kotek, Ali Fuat Yapar, Berrin Pehlivan

**Affiliations:** 1 Baskent University Adana Medical Faculty, Department of Radiation Oncology, Adana, Turkey; 2 Baskent University Adana Medical Faculty, Department of Nuclear Medicine, Adana, Turkey; 3 Akdeniz University Medical Faculty, Department of Radiation Oncology, Antalya, Turkey

**Keywords:** Concurrent chemoradiotherapy, locally advanced pancreas cancer, positron emission tomography, metabolic response, clinical outcome prediction

## Abstract

**Background:**

We aimed to study the predictive value of combined 18F-fluoro-deoxy-D-glucose positron emission tomography and computerized tomography (FDG-PET-CT), on outcomes in locally advanced pancreatic carcinoma (LAPC) patients treated with concurrent chemoradiotherapy (C-CRT).

**Methods:**

Thirty-two unresectable LAPC patients received 50.4 Gy (1.8 Gy/fr) of RT and concurrent 5-FU followed by 4 to 6 cycles of gemcitabine consolidation. Response was evaluated by FDG-PET-CT at post-C-CRT 12-week. Patients were stratified into two groups according to the median difference between pre- and post-treatment maximum standard uptake values (SUV_max_) as an indicator of response for comparative analysis.

**Results:**

At a median follow-up of 16.1 months, 16 (50.0%) patients experienced local/regional failures, 6 of which were detected on the first follow-up FDG-PET-CT. There were no marginal or isolated regional failures. Median pre- and post-treatment SUV_max _and median difference were 14.5, 3.9, and -63.7%, respectively. Median overall survival (OS), progression-free survival (PFS), and local-regional progression-free survival (LRPFS) were 14.5, 7.3, and 10.3 months, respectively. Median OS, PFS, and LRPFS for those with greater (N = 16) versus lesser (N = 16) SUV_max _change were 17.0 versus 9.8 (p = 0.001), 8.4 versus 3.8 (p = 0.005), and 12.3 versus 6.9 months (p = 0.02), respectively. On multivariate analysis, SUV_max _difference was predictive of OS, PFS, and LRPFS, independent of existing covariates.

**Conclusions:**

Significantly higher OS, PFS, and LRPFS in patients with greater SUV_max _difference suggest that FDG-PET-CT-based metabolic response assessment is an independent predictor of clinical outcomes in LAPC patients treated with definitive C-CRT.

## Background

The Gastrointestinal Study Group trial set concurrent chemoradiotherapy (C-CRT) as the standard of care for medically-fit patients with locally advanced pancreatic carcinoma (LAPC) [1]. However, local/regional relapse rates (42-68%) are still unacceptably high [1,2], and may be related to the limited radiosensitizing efficacy of available chemotherapeutics and/or insufficiency of conventionally used radiation doses of 45-50.4 Gy [3]. Additionally, geographic misses, due to target volume delineation difficulties using conventional imaging, may also contribute. For radiotherapy treatment planning (RTP), sensitivity and specificity of contrast-enhanced computerized tomography (CT), the standard method for tumor volume delineation, are insufficient for defining primary tumor boundaries and nodal extensions [4-11], emphasizing the need for novel tools.

18F-fluoro-deoxy-D-glucose positron emission tomography (FDG-PET) provides useful information about tissue metabolism. Studies investigating FDG-PET have demonstrated significantly better sensitivity, specificity, and accuracy rates for FDG-PET over CT, in defining local, regional, and systemic extent of disease in several tumor sites, including the pancreas [6-8,10,12-17]. Further, Delbeke et al. [18] and Lemke et al. [19] demonstrated significantly better rates for FDG-PET and FDG-PET-CT over CT in diagnosing malignancy and determining local/regional extensions in LAPC.

Growing evidence indicates the need for integration of functional tumor/surrounding information into modern RTP practice, to improve target volume delineation. While anatomic restrictions, related to relatively poor spatial resolution, limit FDG-PET use in RTP, such restrictions may be overcome by its co-registration with CT-provided anatomical data [20,21]. Thus, FDG-PET-CT-based RTP studies have resulted in RT field alterations [22-24]. We previously compared CT versus co-registered FDG-PET-CT for gross tumor volume (GTV) delineation, and demonstrated a statistically significant increase in GTV in 35.7% patients, with incorporation of FDG-PET data [3].

FDG-PET has also been demonstrated to have a beneficial role in predicting clinical outcomes [25], albeit in only a few studies of pancreatic carcinoma, and without firm conclusions [9,11,26-29]. Therefore, this prospective study was designed to assess the predictive utility of post-treatment FDG-PET-based metabolic response on clinical outcomes, in medically-fit, unresectable, LAPC patients, treated with C-CRT using co-registered FDG-PET-CT-based RTP.

## Methods

### Patients

Patient eligibility details have been previously reported [3]. Thirty-two patients with unresectable, non-metastatic LAPC with histologic proof of malignancy were prospectively enrolled. Our institutional definition for technically unresectable pancreatic carcinoma is to be stage III (T4N0-1M0) disease, which is the involvement of celiac axis and/or superior mesenteric artery. Disease extent was determined by radiological studies and laparoscopy/laparotomy. Standard radiologic studies included contrast enhanced abdominal CT, magnetic resonance imaging (MRI) and/or MR-cholangiopancreaticography (MRCP). We also restaged patients with FDG-PET-CT obtained for RTP. All eligible patients underwent laparoscopic or laparotomic examination and biopsies for histologic diagnosis of primary tumor and enlarged/metabolically active regional lymph nodes and isolated single organ metastasis respecting the current standard institutional staging procedure for pancreatic carcinoma.

Patient-signed informed consent was obtained and the study design was approved by the Institutional Ethical Committee, in accordance with the Helsinki Declaration and Rules of Good Clinical Practice.

### FDG-PET-CT-based Treatment Planning and Treatment Delivery

FDG-PET-CT was performed, within 10 days prior to treatment, according to institutional protocols [3]. Briefly, patients were immobilized supine with arms up. Lasers (Acuity, Varian Medical Systems, Palo Alto, CA, USA) were used to align and mark patients, to define the coordinate system for RTP and treatment sessions. For FDG-PET-based RTP, the combined FDG-PET-CT system (Discovery-STE 8, General Electric Medical System, Milwaukee, WI, USA) was used. Six-hour fasting blood glucose below 150 mg/dl was verified before intravenous (IV) 370-555 MBq18FDG administration. Patients were left supine in a quiet room during the distribution phase, and combined image acquisition commenced 60 minutes after FDG injection.

An enhanced CT with IV plus oral contrast media through 5-mm slices from the skull base to the pelvis inferior border was acquired using a standardized protocol with 140 kV/80 mA. Thereafter, the PET scan was acquired in 3D mode from skull base to the pelvis inferior border (6-7 bed positions, 3 minutes/position) without repositioning. CT and PET images were acquired with the patient breathing shallowly. Attenuation was corrected using CT images. FDG uptake areas were categorized as malignant by location, intensity, shape, size, and visual correlation with CT images, to differentiate physiologic from pathologic uptake.

Image registration and RTP were performed via the Eclipse 7.5 RTP system (Varian Medical Systems). Two radiation oncologists, assisted by a nuclear medicine physician, defined target volumes and contoured the GTV, planning target volume (PTV), and organs at risk, on the co-registered FDG-PET-CT images. GTV included the primary tumor and involved lymph nodes apparent on CT (short axis ≥ 1.5 cm) and/or FDG-PET images. PTV was defined as GTV+2 cm in each direction, allowing for microscopic extension and setup errors. The primary tumor and involved lymph node(s) alone were irradiated, without elective regional irradiation.

A single target volume, without cone down volumes, using a four-field technique (postero-anterior/antero-posterior/laterals) was irradiated. Treatment volumes were defined using customized multi-leaf collimators. Patients received RT regimens using 18 MV photon energy linear-accelerators. A dose of 50.4 Gy (1.8 Gy/fr, 5 days/week) encompassed the defined PTV with isodose lines not cooler or hotter than 95% and 107%, respectively. To achieve this, dosimetric practice wedges modified beams. Dose-volume histograms assessed patient target volume coverage and organ-at-risk doses. For normal tissues, maximum dose limits were 45 Gy for spinal cord; 50 Gy for small bowel and stomach; 50 Gy for ≤ one-third, 35 Gy for two-thirds, and 30 Gy for three-thirds of the liver; and 20 Gy for at least two-thirds of one functioning kidney. Dose levels beyond these limits were considered as exclusion criteria.

### Chemotherapy

Patients received continuously-infused 5-FU (225 mg/m2/day, 7 days/week) throughout the RT course as a radiosensitizer, and additional maintenance treatment (4-6 course) with gemcitabine (1000 mg/m2 IV over 100 minutes, days 1, 8 and every 21 days) after C-CRT completion.

### Response Evaluation and Follow-up

Treatment response was assessed by re-staging FDG-PET-CT scans from post-C-CRT 12-week follow-up, according to EORTC-1999 guidelines [30]. The time interval of 12-week for the first follow-up FDG-PET-CT was mandatorily chosen as the shortest possible time for response assessment according to our national health insurance politics, rather than an evidence based practice. Thereafter, patients were monitored by 8-12 weekly studies (blood count/chemistry; serum CEA and CA 19-9). Additional abdominal ultrasound and/or CT, chest CT, cranial magnetic resonance imaging, and FDG-PET-CT were used as indicated.

### Statistical Analyses

The primary endpoint was to assess the predictive usefulness of FDG-PET-CT-based metabolic response, following C-CRT, on clinical outcomes by assessing maximum standard uptake value (SUV_max_) differences between pre- and post-treatment scans. Patients were categorized into two groups (response greater versus lesser than the median difference; one-sample T test) and compared by local/regional progression-free survival (LRPFS), progression-free survival (PFS), and overall survival (OS). LRPFS was defined as survival without local/regional failure, calculated as the time between the first day of treatment and the date of local/regional failure or death/last visit. PFS and OS were calculated as the time between the first day of treatment and any type of disease progression, and the date of death/last visit, respectively. Survival analysis was performed using the Kaplan-Meier method, and survival curves were compared with two-sided log-rank tests. Cox proportional hazard model was applied to evaluate the relationship of SUV_max _difference (greater or lower than median) and known prognostic variables, including age (continuous), gender (male or female), ECOG performance statuses (ECOG 0-1 or 2), nodal involvement (N0 or N1), hemoglobin levels (< 12 or ≥ 12 g/dl), CA 19-9 levels (< 100 or ≥ 100), CEA levels (< 10 or ≥ 10), pancreatic primary (head or body), weight loss (≥ 5% or < 5%) with survival. Since the study population is small, and exact prognostic values of these parameters are not clear, we chosen enter selection method. All statistical tests are two-sided, and p < 0.05 was considered statistically significant.

## Results

Forty-four patients were enrolled, and 32 were eligible. Twelve (27.3%) were excluded due to distant metastasis [peritoneal surfaces (N = 5), liver (N = 4), multiple organs (N = 3)] apparent on FDG-PET but not CT, and referred to the Department of Medical Oncology for systemic therapy. Patient, disease, and treatment characteristics of 32 eligible patients were as depicted in Table 1. Patients tolerated C-CRT without grade 3-4 acute toxicity. No chemotherapy dose reduction was necessary, but treatment was interrupted in 3 cases (9.4%) due to intractable grade 3 acute diarrhea for 3, 5, and 5 days, respectively.

**Table 1:** Patient, tumor, and treatment characteristics

Characteristic	Overall (N = 32)	Greater SUV response (%) (N = 16)	Lesser SUV response (%) (N = 16)	p-value
Age (Years)				
Median	58.2	59.3	57.6	0.32
Range	37-69	43-69	37-64	
Gender (%)				0.43
Male	23 (71.9)	11 (34.4)	12 (37.5)	0.43
Female	9 (28.1)	5 (15.6)	4 (12.5)	
ECOG Performance				0.26
ECOG 0-1	24 (75)	11 (34.4)	13 (40.6)	
ECOG 2	8 (25)	5 (15.6)	3 (9.4)	
Hemoglobin				0.22
Median	10.8	10.6	11.1	
Range	9.4-16.2	9.4-15.2	9.7-16-4	
< 12 g/dl (%)	21 (65.6)	11 (34.4)	10 (31.2)	
≥ 12 g/dl (%)	11 (34.4)	5 (15.6)	6 (18.8)	
CA 19-9 (%)				0.58
< 100	7 (21.9)	4 (12.5)	3 (9.4)	
≥ 100	25 (78.1)	12 (37.5)	13 (40.6)	
CEA (%)				0.63
< 10	27 (84.4)	13 (40.6)	14 (37.5)	
≥ 10	5 (15.6)	3 (9.4)	2 (12.5)	
Pancreatic Primary (%)				0.63
Head	27 (84.4)	14 (37.5)	13 (40.6)	
Body	5 (15.6)	2 (12.5)	3 (9.4)	
Nodal stage (%)				0.14
0	13 (40.6)	6 (18.8)	7(21.9)	
1	19 (59.4)	10 (31.2)	9 (28.1)	
Weight loss (%)				0.19
≥ 5%	22 (68.8)	14 (37.5)	10 (31.2)	
< 5%	10 (31.2)	2 (12.5)	6 (18.8)	
SUVmax				0.45
Median	14.5	14.0	15.1	
Range	6.5-22.6	6.5-20.7	9.5-22.6	

**Abbreviations: **CEA, Carcinoembriogenic antigen; ECOG, Eastern Cooperative Oncology Group; SUV, Standard uptake value.

**Figure 1:**
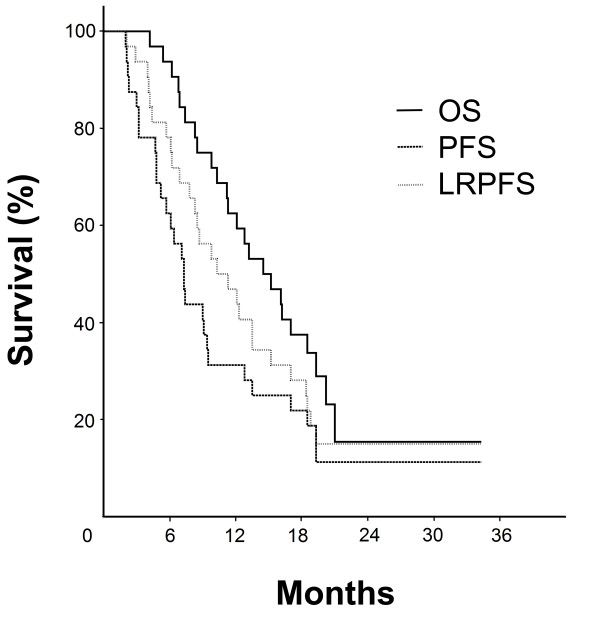
**Survival curves for study population**. Solid line: OS; Dashed line: PFS; Doted line: LRPFS.

At a median follow-up of 16.1 months (range: 4.2-34.3), 24 of 32 patients (75%) were dead. Outcomes of eligible 32 patients were as depicted in Table 2. Median OS, PFS, and LRPFS were 14.5 (95% CI: 9.9-19.1), 7.3 (95% CI: 5.9-8.7), and 10.3 months (95% CI: 5.6-15.0), respectively (Figure 1). Twenty-six patients (81.3%) experienced some failure during the follow-up period. Sixteen patients (50.0%) developed infield recurrences, 3 (9.4%) of which were isolated, and 13 (40.6%) were concomitant with distant relapses. Distant relapses without local failures were encountered in 10 (31.3%) patients, but ultimately distant relapses were evident in 23 patients (71.9%). Distant failure sites included the liver (N = 9), peritoneum (N = 7), and multiple organs (N = 7 patients). There were no marginal or isolated regional recurrences.

**Table 2:** Treatment outcomes of eligible 32 patients

Patient	Pre-PET gemcitabine cycles	Total gemcitabine cycle	Pre-treatment SUV_max_	Post-treatment SUV_max_	Relative SUV change (%)	SUV response (G/L/P)	OS (mo)	LRPFS (mo)	PFS (mo)	Resection status (N/Y)
1	3	5	13,5	1,9	-85,9	G	16,4	16,4	8,4	Y
2	2	6	11,3	2,4	-84,8	G	15,5	15,5	15,5	N
3	4	5	14,9	3,9	-83,8	G	17,0	17,0	17,0	N
4	2	6	10,5	1,5	-83,7	G	16,2	9,2	6,1	Y
5	2	4	13,8	3,4	-80,5	G	14,5	10,3	7,3	N
6	3	6	14,2	2,9	-79,6	G	17,5	17,5	17,5	Y
7	4	6	7,9	2,8	-77,6	G	8,5	8,5	5,2	N
8	3	4	19,2	4,4	-77,1	G	12,8	7,8	4,8	N
9	4	5	16,8	2,4	-75,7	G	14,2	10,5	10,5	N
10	2	4	20,7	3,1	-75,1	G	20,4	20,4	20,4	Y
11	3	4	8,6	1,4	-73,7	G	34,3	34,3	34,3	Y
12	4	5	7,8	2,3	-70,5	G	18,0	12,3	9,0	N
13	4	6	6,5	2,1	-67,7	G	11,3	6,1	6,1	N
14	3	4	14,8	2,3	-64,6	G	17,2	17,2	17,2	N
15	2	4	17,6	3,4	-64,3	G	20,2	10,5	7,5	Y
16	3	5	15,8	5,7	-63,9	G	15,2	15,2	7,4	N
17	4	5	17,3	2,5	-63,6	L	18,8	18,8	12,8	N
18	2	4	10,4	4,1	-61,0	L	16,1	11,3	6,4	N
19	4	4	22,6	3,9	-56,7	L	11,2	5,7	5,7	N
20	3	5	18,6	4,2	-55,4	L	25,8	25,8	19,3	N
21	3	5	15,2	3,9	-54,3	L	16,3	16,3	16,3	N
22	4	6	12,2	6,8	-54,3	L	9,8	9,8	3,2	N
23	4	5	16,9	4,1	-52,8	L	8,3	8,3	4,7	N
24	4	4	15,7	8,1	-48,4	L	10,3	4,1	3,8	N
25	2	4	12,4	5,8	-43,2	L	10,3	10,3	7,3	N
26	3	5	11,5	8,7	-34,3	L	13,2	8,7	7,3	N
27	2	4	15,1	20,4	25,1	P	7,4	4,4	2,3	N
28	4	5	9,5	13,2	38,9	P	6,8	4,0	3,0	N
29	2	4	11,5	18,7	41,6	P	4,2	4,2	3,2	N
30	3	5	16,2	21,4	42,1	P	6,9	6,9	2,2	N
31	4	4	16,3	24,1	47,8	P	5,4	2,9	2,0	N
32	3	5	11,2	17,3	54,5	P	6,2	2,9	2,1	N

**Abbreviations: **G, Greater; L, Lesser, LRRFS, Local-regional progression free survival; Mo, Months; N, No; OS, Overall survival; P, Progression; PFS, Progression free survival; SUV_max_, Maximum standard uptake value; Y, Yes.

**Figure 2:**
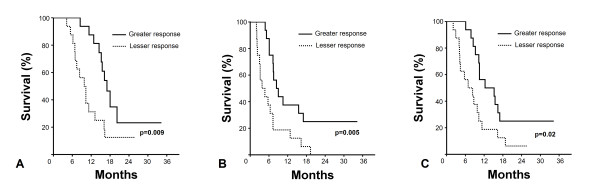
**Comparative survival analyses between patients with greater and lesser PET-CT response**. A: Overall survival (OS); B: Progression-free Survival (PFS); C: Local Regional Progression-free Survival (LRPFS). Solid line: greater SUV_max _change; Dashed line: lesser SUV_max _change.

Median pre- and post-treatment SUV_max _levels were 14.5 (range: 6.5-22.6) and 3.9 (range: 1.4-24.1), respectively. Response evaluation at the 12-week FDG-PET-CT follow-up revealed increased metabolic activity in 6 (18.7%) and decreased in 26 (81.3%) cases. Median SUV_max _difference was -63.7% (range -85.9 - 54.5). Comparative survival analysis revealed a statistically-significant superiority for patients depicting a SUV_max _reduction greater than 63.7% in terms of OS, PFS, and LRPFS (Figure 2). Corresponding median survival times for the patient group with greater versus lesser SUV_max _change were 17.0 (95% CI: 14.5-19.4) versus 9.8 (95% CI: 7.2-12.4) for OS (p = 0.009), 8.4 (95% CI: 5.5-11.3) versus 3.8 (95% CI: 1.8 - 6.7) for PFS (p = 0.005), and 12.3 (95% CI: 3.1-21.5) versus 6.9 months (95% CI: 1.8-12.0) for LRPFS (p = 0.02), respectively. There were no significant difference between two groups in terms of patient, disease, and treatment related factors, which may potentially impact prognosis (Table 1). Multivariate analysis based on these factors demonstrated that, only the SUV response retained its independent prognostic value on OS (p = 0.007), PFS (p = 0.008), and LRPFS (p = 0.018), respectively.

Another factor anticipated to potentially alter both SUV_max _change and treatment outcomes was the average cycles of gemcitabine received by patients in each response group. Analysis revealed that patients in greater versus lesser metabolic response groups received 3.0 (range; 2-4) and 3.2 cycles (range; 2-4) prior to 12 week PET-CT scan (p = 0.74), and a total of 4.9 (range; 4-6) and 4.6 cycles (range; 4-6) of gemcitabine (p = 0.62), respectively. Further analysis revealed that the OS, PFS, and LRPFS advantage of the group with greater SUV change over that with lesser SUV change was also independent of gemcitabine chemotherapy received by patients (p = 0.009, p = 0.013, and p = 0.024, respectively).

**Figure 3:**
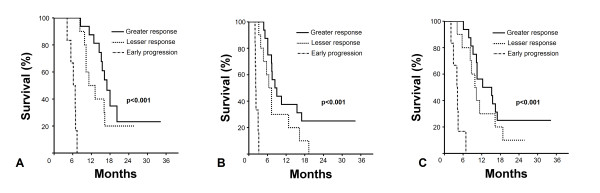
**Comparative survival analyses between patients with greater PET-CT response, lesser PET-CT response and early progression**. A: Overall survival (OS); B: Progression-free Survival (PFS); C: Local Regional Progression-free Survival (LRPFS). Solid line: greater response; Doted line: lesser response; Dashed line: early progression.

6 of 16 patients (37.5%) in group with lesser metabolic response demonstrated early disease progression at 12-week PET-CT evaluation. To analyze the value of this findings on outcomes we further stratified patients into 3 respective groups; Group 1: Greater SUV response (N = 16); Group 2: Lesser SUV response (N = 11); and Group 3: Early progression. Corresponding median survival times in Groups 1 vs. 2 vs. 3 were 17.0 (95% CI: 14.5-19.4) vs. 11.2 (95% CI: 6.7-15.7) vs. 6.2 (95% CI: 4.5-7.9) for OS (p < 0.001); 8.4 (95% CI: 5.5-11.3) vs. 6.4 (95% CI: 4.7-8.1) vs. 2.2 (95% CI: 2.0-2.4) for PFS (p < 0.001); and 12.3 (95% CI: 3.1-21.5) vs. 9.8 (95% CI: 7.3-12.3) vs. 4.0 (95% CI: 2.4-5.6) for LRPFS (p < 0.001), respectively (Figure 3).

Analysis of the impact of metabolic response on tumor resectability following C-CRT demonstrated that 6 of 16 patients (37.5%) with higher SUV response became resectable compared to none among those who progressed early (N = 6) or responded lesser than -63.7% (N = 10). At the time of this analysis, 4 of the 6 resected patients (66.7%) were still alive at 18.8, 20.4, 25.8, and 34.3 months of follow-up, while remaining two patients were lost due to widespread disease progression at 18.0 and 20.2 months, respectively.

## Discussion

Compared to conventional contrast-enhanced CT, both FDG-PET and FDG-PET-CT are superior for malignancy diagnosis and local extension determination in PC [9,18,19,31]. In patients with PC, Delbeke et al. [18] showed higher diagnostic sensitivity, specificity, and accuracy for FDG-PET over CT (92%, 85%, and 91% versus 65%, 61%, and 65%, respectively). In a larger cohort of 104 patients, Lemke et al. [19] reported that FDG-PET-CT improved malignancy detection sensitivity from 76.6% (CT) and 84.4% (FDG-PET) to 89.1%. In their literature review, Pakzad et al. [32] also described good sensitivity (90-95%) and specificity (82-100%) of FDG-PET for PC detection. In a recent meta-analysis by Tang et al. [33], including 51 studies, sensitivity of FDG-PET-CT (90.1%) was significantly higher than FDG-PET (88.4%) and endoscopic ultrasonography (81.2%). Taken together, these suggest potentially improved diagnosis, staging, and treatment of PC via FDG-PET-CT.

An important contribution of FDG-PET in PC management is its potential to alter planned treatments by detecting CT-occult distant metastasis. In our study, addition of FDG-PET to CT data upstaged 12 (27.3%) of 44 patients from stage 3 to 4, and changed treatment intent from curative to palliative. Therefore, more than one-fourth of patients were spared useless and potentially toxic C-CRT, and instead referred to systemic treatment without delay. Supporting this observation, a study by Delbeke et al., of 21 patients with stage 4 PC, demonstrated that metastases were diagnosed both on CT and FDG-PET in only 10 (47.6%) of 21 patients, but FDG-PET detected distant metastases, not identified on CT, in 7 (33.3%) additional patients [34]. Similarly, Mertz et al. [35], demonstrated that FDG-PET detected 7 (77.8%) of 9 proven metastases, while CT only detected 3 (33.3%). Taken together, these indicate the importance of accurate staging for precise management of such patients.

The first step in tumor control rate improvement with RT is accurate definition of the primary tumor and its local/regional extensions. However, relatively low sensitivity and specificity of conventional imaging techniques makes attaining this goal difficult. Lemke et al. [19] demonstrated higher sensitivity of FDG-PET-CT over CT in depicting adjacent tissue invasion (47.7% versus 68.2%), highlighting the weakness of CT in delineating tumor burden, which is important for RT success. Results of our earlier study revealed that an average 29.7% enlargement in GTV was necessary in 5 of 14 (35.7%) patients because of CT-occult additional lymph node metastases and/or primary tumor extensions detected by FDG-PET-CT [3]. Based on these results, FDG-PET-CT was used for target volume definition in the current study.

Despite its accordance with the CT-based C-CRT literature, reported range of 42-68% [1,2], the 50% infield failure rate, observed here, contrasts with our expectations. Rather than due to inefficacy in accurate target volume definition, failures may be related to other reasons. Limited radiosensitizing efficacy provided by 5-FU may have contributed. Gemcitabine, with its strong radiosensitizing properties, is promising [36-40], but impact of its concurrent use on treatment outcomes remains to be investigated in the era of metabolic response assessment. Another contributor may be the potential insufficiency of 50.4 Gy RT. Unfortunately, results of dose escalation studies failed to report an advantage for higher doses [1,41,42]. However, based on our observation of no marginal and regional failures, and on > 90% local control rates achieved in stereotactic body RT studies [43-45], testing concurrent gemcitabine and escalated RT doses by using "dose-painting" RT, after mapping involving site metabolic activity, may be useful.

FDG-PET-based metabolic response assessment following anticancer therapies has been reported as a strong predictor of clinical outcomes [25]. However, in the era of PC, there are reports with conflicting results [6,9,11,26-29,46,47]. To our knowledge, despite its small size, our study is unique in that it used the SUV_max _difference as a tool for assessing response following definitive C-CRT in patients with LAPC. Here, compared to baseline values, SUV_max _was decreased in 26 (81.3%) patients, and SUV_max _difference > 63.7% was associated with significantly improved OS, PFS, and LRPFS. These results are in line with those of similar studies. Choi et al. [26] demonstrated that patients with FDG-PET response ≥ 50% (N = 2) had better surgical resection and OS compared to those with < 50% (N = 16). Likewise, in our study, 6 of 16 patients (37.5%) with higher SUV_max _response became resectable, compared to none of those with lesser response (N = 10). In a study with 9 cases treated with neoadjuvant CRT, Rose et al. [9] found that all four patients with ≥ 50% reduction, and only 2 of 5 patients with lesser response, were able to undergo resection. Another study of 10 PC patients treated with arterial chemo-infusion and RT [27] showed that FDG-PET aided in assessing the efficacy of treatment over CT in 4 patients. In 2 patients, only FDG-PET detected a therapeutic response, and in the other 2 patients, FDG-PET showed a therapeutic response before CT detected tumor size changes. Bang et al. [11] reported response evaluation by both FDG-PET and CT scans in 15 patients with CRT-treated PC. Six patients, with > 50% reduction in FDG uptake, had longer time to tumor progression. In this regard, our current data not only strongly support the available literature, but also suggest an independent role for "relative change in SUV_max _values" in predicting clinical outcomes following C-CRT.

## Conclusions

Although limited by small sample size, our results revealed two major findings. First, accordant with available literature, we showed that integration of FDG-PET-CT into LAPC management has the potential to alter planned treatments by detecting CT-occult metastasis in 27.3% of patients. Therefore, by using FDG-PET-CT scanning, more than one-fourth of patients may be spared useless and potentially futile CRT protocols. And second, significantly higher OS, PFS, and LRPFS in patients with greater SUV response suggest "metabolic response assessment" as an independent predictor of clinical outcomes in LAPC. In conclusion, despite these positive findings, our current results should be accepted as a baseline, rather than a guide, and be validated by future larger studies.

## Competing interests

We have no personal or financial competing interest and have not entered into any agreement that could interfere with our access to the research data, or upon our ability to analyze the data independently, to prepare manuscripts, or to publish them.

## Authors' contributions

Study conception and design: ET. Provision of study materials or patients: ET, AK, CP. Collection and assembly of data: ET, AK, CP. Data analysis and interpretation: ET, CP, BP, AFY. Manuscript writing: ET, CP.

Final approval of manuscript: ET, CP, AK, AFY, BP.

## Pre-publication history

The pre-publication history for this paper can be accessed here:

http://www.biomedcentral.com/1471-230X/11/123/prepub
